# Analysis of genetic polymorphism of methylenetetrahydrofolate reductase in a large ethnic Hakka population in southern China

**DOI:** 10.1097/MD.0000000000013332

**Published:** 2018-12-14

**Authors:** Pingsen Zhao, Jingyuan Hou, Hesen Wu, Miaocai Zhong

**Affiliations:** a Clinical Core Laboratory; b Center for Precision Medicine, Meizhou People's Hospital (Huangtang Hospital), Meizhou Hospital Affiliated to Sun Yat-sen University; c Guangdong Provincial Engineering and Technology Research Center for Molecular Diagnostics of Cardiovascular Diseases; d Meizhou Municipal Engineering and Technology Research Center for Molecular Diagnostics of Cardiovascular Diseases, Meizhou, P. R. China.

**Keywords:** genetic polymorphism, Hakka population, methylenetetrahydrofolate reductase, southern China

## Abstract

Methylenetetrahydrofolate reductase (*MTHFR*) catalyzes conversion of methylene tetrahydrofolate to methylte trahydrofolate. *MTHFR* C677T polymorphism has been regarded as a risk factor for various vascular diseases. Our study aimed to investigate the distribution frequencies of this polymorphism among Hakka population living in southern China. We retrospectively recruited 5102 unrelated Chinese Hakka subjects. *MTHFR* C677T polymorphism was tested using the polymerase chain reaction (PCR) and DNA sequencing. A total of 2358 males and 2744 females (aged from 10 years to 101 years) were included in this study. In total, 2835 (55.63%) subjects were homozygous for the C allele (CC), 1939 (38.00%) subjects were heterozygous (CT), and 325 (6.37%) subjects were homozygous for the T allele (TT). The allelic frequency of mutant T was 25.37% with 325 individual homozygous for this defective allele resulting in a frequency of about 6.37% for the TT genotype. According to the study results, the overall frequency of *MTHFR* C677T genotypes did not differ significantly among the gender and age groups. Our study showed the prevalence of *MTHFR* C677T polymorphism in a large ethnic Hakka population living in southern China. It would be important implications for the primary prevention of various vascular diseases.

## 1 Introduction

The 5,10-methylenetetrahydrofolate reductase (*MTHFR*) gene, a folate-dependent enzyme that catalyzes the irreversible conversion of 5,10-methylenetetrahydrofolate to 5-methyltetrahydrofolate, thereby directing folate metabolites towards the DNA methylation pathway from the DNA synthesis pathway.^[1–3]^ The *MTHFR* gene has been mapped to the end of the short arm of chromosome 1p36.3 and at least 2 significant functional polymorphisms of the *MTHFR* gene, C677T, and A1298C, have been identified.^[4,5]^ The most common polymorphism in the gene encoding the catalytic domain of *MTHFR*, namely there is a cytosine (C) to thymine (T) substitution at nucleotide 677 in exon 4, which subsequently leads to an alanine-to-valine conversion at amino acid 222 in the *MTHFR* protein, with eventual reduction or loss of *MTHFR* activity.^[6–8]^ Individuals with the 677TT genotype, have approximately 30% the *MTHFR* enzyme activity as compared with those with the 677CC genotype, whereas heterozygotes 677CT have around 65% of enzymatic activity.^[9–12]^ A large number of epidemiological studies have investigated the relationship between the C677T polymorphism of *MTHFR* and many diseases, including breast cancer, colorectal cancer, ischemic stroke, depressive disorders, and hypertension.^[13–17]^

Hakka is one of intriguing Chinese Han population that mainly inhabit in southern China and also widely distribute in Southeast Asia. With a total area of 15,876 square kilometers and a population of 5.43 million, the Meizhou region is located in the northeast of Guangdong Province, China. It is noteworthy that more than 95% of people who live in this area are Hakka and exhibited lots of unique features including in dialects, life styles, intra-ethnic marriages, customs, and architecture.^[18]^ Previous studies have revealed that the prevalence of *MTHFR* C677T is different in distinct geographical areas, races and ethnic populations.^[19–22]^ However, polymorphism of *MTHFR* in the Hakka population remains unclear. Therefore, the aim of the present study was to examine the distribution frequencies of *MTHFR* C677T polymorphism in the Hakka populations in southern China.

## 2 Material and methods

### 2.1 Population sample

**Figure 1:**
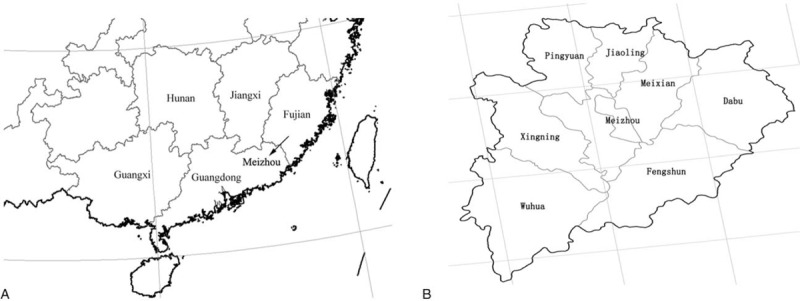
The geographical location and surrounding area of Meizhou area. A: The Meizhou region in southern China. B: The administrative region of Meizhou.

We retrospectively recruited 5102 unrelated Chinese Hakka subjects who visited Meizhou People's Hospital (Huangtang Hospital), Meizhou Hospital Affiliated to Sun Yat-sen University, Guangdong Province, China, between July 2015 and October 2017. All subjects were from families who had been living in the Meizhou area for at least 3 generations of Hakka paternal ancestry. The geographical location of the sampled area is shown in Figure 1. All participants for the study signed written informed consent form according to the ethical guidelines of the Helsinki Declaration before their enrollment. The study was approved by the Committee of Ethics and Research of the Meizhou People's Hospital for experiments involving humans.

### 2.2 DNA extractions

Three millilitres of venous blood was sampled from each subject and collected into tubes containing ethylene diamine tetra-acetic acid (EDTA). Genomic DNA extraction was carried out using Puregene Blood Core Kit C (Qiagen, Germantown, MD) following the manufacturer's instructions and quantified using Nanodrop 2000 Spectrophotometer (Thermo Scientific, DE). The extracted DNA was dissolved in sterile distilled water and stored at −20°C until the day of analysis.

### 2.3 Polymerase chain reaction (PCR) and DNA direct sequencing

**Figure 2:**
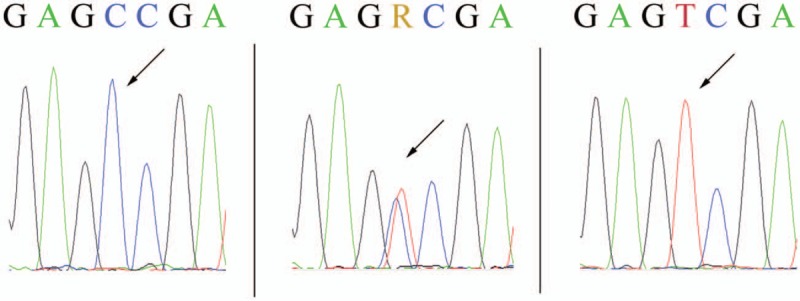
Sequence chromatogram of *MTHFR* C677T gene polymorphism (a) genotype 677CC, (b) genotype 677CT, (c) genotype 677TT.

Detection of the C677T polymorphism of *MTHFR* was performed using a PCR and DNA direct sequencing method according to the manufacturer's instructions (SinoMDgene Technology Co., Ltd., Beijing, China). Briefly, the initiated PCR were amplified in a total volume of 25 uL, containing 20 ng DNA, and the recommended amounts of dNTPs, primers and of Hotstar Taq DNA polymerase. For PCR, an initial denaturation step at 95°C for 3 minutes. was followed by 45 cycles of denaturation at 94°C for 15 seconds, annealing and extension at 60°C at the indicated temperature for 45 seconds, followed by a final extension step of 25°C for 1 min. The PCR products were then purified and for sequenced by dye termination sequencing using BigDye Terminator Cycle Sequencing V3.1 Kit and 3500Dx DNA Analyzer 5.4 (Applied Biosystems, CA). DNA sequences were assembled using ABI PRISM sequencing analysis software version 5.4 (Applied Biosystems, CA). Additionally, to control for correct sample handling, genotyping was randomly repeated in 10% of the samples, and no discrepancies were detected in all repeated experiments when compared with the initial genotyping. The chromatograms of different single nucleotide polymorphism variants using reverse primers for sequencing are presented in Figure 2. The observed genotype frequencies of C677T were compared with expected genotype frequencies according to the Hardy–Weinberg law.

### 2.4 Statistics

The statistical package for the social sciences software (version 19.0; SPSS Inc., Chicago, IL) was used for data all statistical analysis. Genotype and allele frequency differences between groups were assessed using Chi-square and Fisher exact tests. Probability values less than .050 were considered statistically significant.

## 3 Results

A total of 5102 subjects (2358 males and 2744 females, aged from 10 years to 101 years), from a Hakka population living in the city of Meizhou, in southern China were included in this study. Genotype and allelic frequencies of the C677T polymorphism of *MTHFR* according to gender and age are presented in Table 1. No significant departures from Hardy–Weinberg equilibrium was observed for the frequencies of alleles and genotypes of C677T in the Hakka population (*χ*^2^ = 0.06, *P* >.05). In total, 2835 (55.63%) subjects were homozygous for the C allele (CC), 1939 (38.00%) subjects were heterozygous (CT), and 325 (6.37%) subjects were homozygous for the T allele (TT). The allelic frequency of mutant T was 25.37% with 325 individual homozygous for this defective allele resulting in a frequency of about 6.37% for the TT genotype. According to the study results, the overall frequency of *MTHFR* C677T genotypes did not differ significantly among the gender and age groups.

**Table 1:**
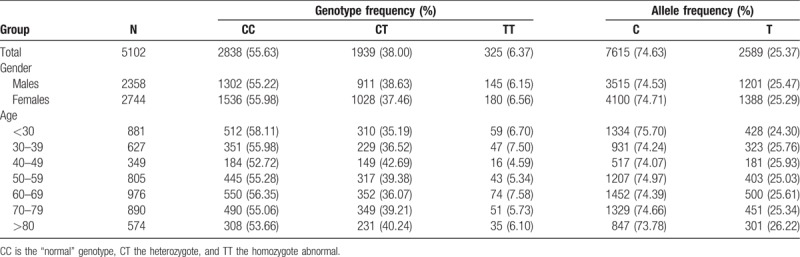
Genotypes and alleles frequencies of *MTHFR* C677T in the Hakka population.

We compared genotype and allele frequencies between our data and previously published data from different countries and ethnic groups worldwide, as shown in Table 2. We further compared the genotype and allele frequencies estimated here for *MTHFR* C677T with respect to previously studied population from different regions and ethnic in China (Table 3). Our results showed that the frequency of *MTHFR* C677T was significantly different according to the geographic region and the ethnicity of the population.

**Table 2:**
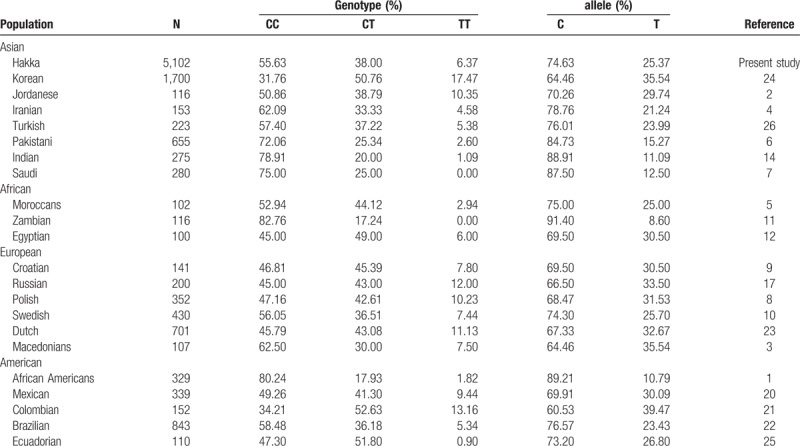
Allele frequencies of *MTHFR* C677T polymorphisms among the Hakka ethic population and other previously studied populations worldwide.

**Table 3:**
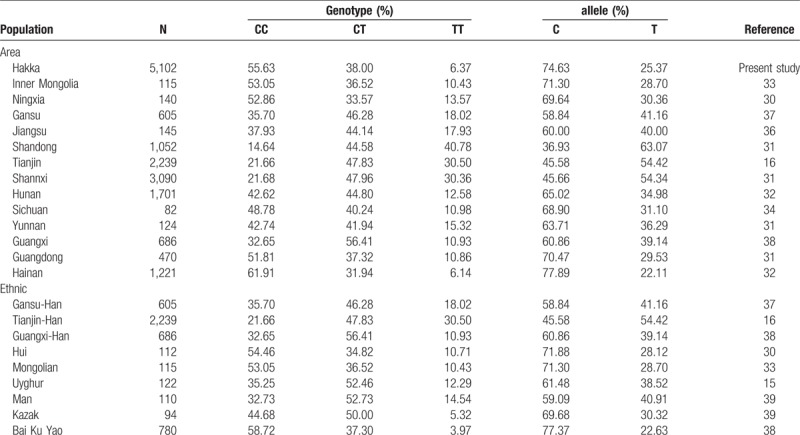
Allele frequencies of *MTHFR* C677T polymorphisms among the Hakka ethic population and other previously studied populations in China.

## 4 Discussion

Methylene tetrahydrofolate reductase is an important enzyme involved in the conversion of methylene tetrahydrofolate to methylte trahydrofolate, which affects DNA methylation and synthesis.^[1,23]^ Previous studies demonstrated cytosine to thymidine (C > T) base change at nucleotide position 677 of the *MTHFR* gene is often regarded as a risk factor for a wide range of serious diseases and *MTHFR* C677T polymorphisms were distributed by ethnicity.^[10,24]^ To our knowledge, we firstly analyzed the frequency of *MTHFR* C677T polymorphisms in the Hakka population in the world. We compared these results with that of other region and racial populations worldwide. Analysis of the study group for the *MTHFR* C677T polymorphism indicated that 55.63% of individuals were homozygous for the wild-type (CC), 38.00% were heterozygous (CT), and the remaining (6.37%) were homozygous for the mutant allele (TT). The identification of this polymorphism in the Hakka population as a health risk factor will help people who are predisposed to susceptible disease to make adequate health decisions in order to delay or halt the disease.

The homozygous 677TT genotype of this mutation has been found to specify a variant enzyme with reduced activity and to be associated with elevated plasma levels of total homocysteine (tHcy), particularly in the setting of low folate levels, as compared to the wild-type (677CC) and heterozygous (677CT) genotypes.^[25,26]^*MTHFR* C677T polymorphism has been extensively investigated and considered to be a risk factor for various diseases, such as cancer, inflammatory bowel diseases, ischemic stroke, and coronary artery disease.^[27–30]^

The ethnic difference in *MTHFR* C677T polymorphism is well known. Some studies revealed that homozygous state of *MTHFR* C677T polymorphism is variable in populations with certain ethnic backgrounds from Asia, Africa, Europe, and America.^[10,11,19,22]^ Homozygous 677TT were rare or completely absent in African and the frequency of the homozygous wild-type 677CC genotype is more prevalent in Asian and European Caucasian.^[7,9,17,24,25]^ The *MTHFR* 677TT genotype affects an estimated 10% of individuals worldwide (range from 0%–30%). These studies of *MTHFR* gene polymorphisms showed a considerable heterogeneity from country to country, and similar results were also observed among Chinese populations. The previous studies revealed the frequency of homozygous 677TT is high in residents in Shandong (40.78%), Tianjin (30.50%) and Shanxi (30.36%) of China.^[31]^ While in our study the frequency of homozygous 677TT is found to be 6.37% in the Hakka population, similarly 6.14% in residents in Hainan of China.^[32]^ In addition, inconsistent of rates of homozygous 677TT carriers were found in residents of Inner Mongolia (10.43%), Jiangsu (17.93%), Sichuan (10.98%) and Guangxi (14.5%) of China.^[33–38]^ Generally, these results show that the frequency of homozygous mutation genotype of 677TT in northern cities is higher than that in southern cities of China. In the current study, *MTHFR* C677T gene polymorphisms were compared with published data of different ethnic groups in Chinese population. Chinese-Han in Tianjin exhibited the highest proportion of frequency of the 677TT genotype (30.50%).^[16]^ The relatively lower frequency of the 677TT genotype (6.37%) was found in our group, as well as in Kazak (5.32%) and Bai Ku Yao (3.97%) national minority were observed when compared with the frequencies reported in other Chinese study populations.^[38,39]^

Ample evidence has implicated the importance of *MTHFR* gene in the onset and progression of various disorders, particularly those associated with folate and homocysteine status.^[40,41]^ Interestingly, identification of genetic predisposition may hold promises in the development of strategies for personalized risk prediction and strategic health-care planning and substantial studies have revealed an interaction between lifestyle factors and *MTHFR* polymorphisms worldwide. For example, in 2010, Songserm et al performed a nested case–control study in the Thailand population and the result strongly suggest that polymorphisms in *MTHFR* genotypes act together with alcohol drinking and low folate intake to increase the risk of cholangiocarcinomas.^[42]^ Di Daniele et al have explored the effects of Italian Mediterranean organic diet versus low-protein diet in nephropathic patients according to *MTHFR* genotypes. The study result showed that a significant reduction of tHcy in T allele carriers after Italian-style Mediterranean diet and in both genotypes after Italian Mediterranean organic diet subsequently might lead to a lower incidence of cardiovascular disease and could be of particular significance in chronic kidney disease patients.^[43]^ Also, folate and homocysteine were implicated in human reproductive health and, in particular, subfertility. A previous literature revealed significant folate deficiency and higher homocysteine in mediterranean population carrying the *MTHFR* TT genotype, thus emphasizing that periconceptional supplementation combined with the food-based approach or supplement.^[44,45]^ In the present study, we investigate the distribution frequencies of *MTHFR* C677T polymorphism among Hakka population living in southern China. Given these genetic differences, genome-based nutritional advice might be tailored in a regionalized and individualized manner that may lead to a healthier dietary pattern.

Several limitations of the present study deserve to be stressed. First, all the subjects were recruited from hospital, therefore there was a potential selection bias. Second, the present study is lacking information on serum folate and tHcy status. Third, the association between *MTHFR* polymorphisms and risk of disease was not observed.

## 5 Conclusions

Our study is the first report on *MTHFR* C677T polymorphisms in a large Hakka population in southern China. It would be important implications for the primary prevention of various vascular diseases.

## Acknowledgments

The author would like to thank other colleagues who were not listed in the authorship of Clinical Core Laboratory and Center for Precision Medicine, Meizhou People's Hospital (Huangtang Hospital), Meizhou Hospital Affiliated to Sun Yat-sen University for their helpful comments on the manuscript.

## Author contributions

Pingsen Zhao conceived and designed the experiments; Jingyuna Hou and Pingsen Zhao recruited subjects and collected clinical data. Jingyuna Hou, Hesen Wu and Miaocai Zhong conducted the laboratory testing. Pingsen Zhao and Jingyuna Hou prepared the manuscript.

**Conceptualization:** Pingsen Zhao.

**Data curation:** Pingsen Zhao, Jingyuan Hou, Hesen Wu, Miaocai Zhong.

**Formal analysis:** Pingsen Zhao.

**Funding acquisition:** Pingsen Zhao.

**Investigation:** Pingsen Zhao.

**Methodology:** Pingsen Zhao, Jingyuan Hou, Hesen Wu, Miaocai Zhong.

**Project administration:** Pingsen Zhao.

**Resources:** Pingsen Zhao.

**Software:** Pingsen Zhao.

**Supervision:** Pingsen Zhao.

**Validation:** Pingsen Zhao, Jingyuan Hou.

**Visualization:** Pingsen Zhao.

**Writing – original draft:** Pingsen Zhao, Jingyuan Hou.

**Writing – review & editing:** Pingsen Zhao.
